# Virtual classes during COVID-19 pandemic: focus on university students’ affection, perceptions, and problems in the light of resiliency and self-image

**DOI:** 10.1186/s40862-022-00144-7

**Published:** 2022-07-08

**Authors:** Elham Assi, Mojgan Rashtchi

**Affiliations:** Islamic Azad University, North Tehran Branch, Abbasspour Blvd., Hakimiyeh, Tehran, Iran

**Keywords:** Affection, Online learning, Problems, Self-image, Resiliency, Virtual courses

## Abstract

Although online learning has been studied extensively over the last two decades, students’ feedback toward such classes during the COVID-19 pandemic seems essential due to their effects on the education systems. The present exploratory sequential mixed methods study aimed to address university students’ affection, perception, and problems in online courses during the pandemic considering their resiliency and self-image. A 37-item virtual class Affection Perceptions, Problems, Resiliency, and Self-image Questionnaire (APPRSQ) was developed, validated, and administered online to 252 university Translation and TEFL-major students. The results demonstrated that the APPRSQ is a valid and reliable data gathering instrument. The participants’ answers to APPRSQ indicated that they were resilient and could adapt to the new learning mode. However, they preferred face-to-face classes, though they believed online courses were inevitable during the pandemic. Such courses had some advantages, such as enhancing students’ technological knowledge and helping them become autonomous. The triangulated data obtained from interviews with 20 students were analyzed. The results could provide a further understanding of APPRSQ factors. The study suggests that teachers and educational authorities improve the quality of online classes.

## Introduction

Technology has influenced humans’ lifestyles during the last two decades and has caused a dramatic change in teaching and learning. Educational technology as a paradigm has altered the traditional concept of learning (Sun et al., 2008). By the mid-80 s, electronic learning (e-learning) was a subset of distance learning; however, with the advent of the internet, distance learning appeared as an option to facilitate teaching and learning for many individuals (Kanuka & Anderson, 2007).

The sudden outbreak of COVID-19 challenged the education system worldwide and forced virtual learning as a panacea in times of crisis. Most teachers and students participated in virtual courses; however, many believed in the prominence of face-to-face classes. Several reasons were responsible for such preference, such as the slow internet connection, computer bugs, power outages, and handling errors that impacted the teaching and learning process. The risk of students’ inequality and teachers’ knowledge of computers was another source of the problem. Besides, students’ level of engagement in such courses was debatable (Rashtchi & Khoshnevisan, 2021). Despite all limitations, most schools and universities shifted toward virtual learning (Sintema, 2020) since it was the only supplement that could deal with school closure loss due to the pandemic. However, such a change altered the standard concepts regarding educational systems worldwide (Khoshnevisan & Alfahad, 2021).

Thus, exploring different aspects of virtual courses and their impacts on various groups involved in teaching and learning, such as teachers, students, practitioners, and syllabus designers, is necessary and should be the concern of researchers in the field. One reason is that learning outcomes show themselves in a long process after students have invested significant time and effort in such classes. Second, it is essential to examine the challenges of online learning because even if the world overcomes the so-called pandemic, the probability of such occurrences is always with human beings. Third, educators can discover to what extent educational systems worldwide can rely on virtual learning.

The present study investigated the usefulness of online courses during the pandemic from Iranian university students’ perspectives, mainly focusing on their affection, perceptions, and problems. Affective factors are decisive since they include emotional experiences that reflect how an individual becomes aware, interprets, and emotionally links to a context (Mahn & John-Steiner, 2012). The affective domain merges various factors vital for successful learning, such as feelings, emotions, attitudes, motivations, and values (Van Der Hoeven Kraft et al., 2011). Thus, the researchers assumed that investigating students’ affection could show how they successfully adapted themselves to the virtual context during the pandemic. Besides, researchers in the current study presumed that affection as a factor in boosting achievement (Ozel et al., 2012) deserves special attention.

Another factor that the present study focused on was students’ perceptions, which could indicate their physiological reaction to the cognitive contact with the new environment (Efron, 1969). It was necessary to explore how the participants perceived and reacted to the obligation to participate in online classes. The third factor, problems learners encountered in virtual courses, was also crucial since they could hinder the teaching–learning process (Favale et al., 2020) and lead to the breakdown of the educational system.

Further to the focal issues of the present study, learning about the respondents’ resiliency and self-image was essential because such characteristics affect individuals’ reactions to virtual classes. As Waxman et al. (2002) argue, resilience explains why some students do well while others from similar socioeconomic backgrounds and abilities do not. They assert that examining self-image would help understand learners’ flexibility to accept different contexts and thus is a factor in shaping their views. Rodríguez-Fernández et al.’s study (2018) indicated that resilience and personal well-being are decisive psychological variables in predicting adolescent learners’ school engagement and perceived performance. They found that cultivating resilience and subjective well-being improves academic achievement.

### Literature review

The priority of using virtual environments over traditional ones has several rational causes and objectives. Different studies have pointed to various advantages of online education. One advantage is that it removes boundaries, allows students to learn without time and place constraints, and facilitates ubiquitous learning (Ashar et al., 2020). Besides, virtual classes foster interactive activities (Kim & Bennekin, 2013), enhance motivation (Islam et al., 2018), and reduce anxiety (Bakar et al., 2013; Yaniafari & Rihardini, 2021). Aydin (2008) argues that e-learning might enhance students’ interaction due to the motivation and encouragement created by online applications. Additionally, students realize their confidence by directing and controlling their learning (Coverdale-Jones, 2000).

In the same vein, lack of face-to-face contact, which does not let students learn from facial expressions and body language, is another advantage of virtual classes. That is why learners try to use their abilities and resources to learn, leading to autonomous learning (Hall, 2011). Lack of face-to-face interactions also encourages students to think more as part of a community. Therefore, one advantage of virtual classes can be cultivating critical thinking skills (Rashtchi, 2007), an issue neglected in face-to-face courses. Besides, online courses develop student-centered classes where learners build knowledge via interaction with peers and teachers (Boettcher & Conrad, 2010). Since virtual settings are asynchronous, learners’ engagement, motivation, interaction, and feedback are necessary. Despite the advantages of virtual classes over face-to-face ones, holding such classes to reach education goals should be considered in light of students’ emotional states.

Transition to online learning in the COVID-19 era was necessary (Bao, 2020) with its challenges and merits (Usak et al., 2020). Due to lockdown regulation, students mainly suffered from contacting their teachers and studying at school. However, the pandemic forced the education system to look for alternative ways of schooling and instruction (Sintema, 2020). As Mulenga and Marban (2020) argue, COVID-19 opened a new horizon to e-learning and increased the use of social media platforms like Facebook and WhatsApp by thousands of students in K-12 levels and beyond. Employing e-learning as a remedy for the impacts of the pandemic on the education system also increased the use of mobile devices to access lessons and fostered involvement in self-regulated and autonomous practices of teachers and learners (Al-Emran et al., 2016).

Many studies have shown the practicality of e-learning in recent years. For example, Al-Adwan et al. (2013) investigated 107 students’ acceptance of e-learning systems at universities in Jordan. The case study adopted quantitative data collection methods to examine the factors that affect the acceptance of e-learning. The results revealed learners’ willingness to adopt e-learning systems. They also confirmed that learning technologists are user-friendly and enhance learners’ performance.

In another study, Pilli et al. (2014) investigated factors affecting students’ attitudes towards using the e-learning system. The results revealed that the participants were satisfied with using the e-learning system. The findings suggested enhancing the faculty members’ awareness of the necessity of integrating e-learning within the educational process. Further, in their study on learners’ attitudes, Ramadan et al. (2019) found that the participants had a positive attitude toward e-learning. They also found that students’ attitudes differed due to their academic discipline, experience using Information and Communication Technology (ICT) tools, and English proficiency.

In more recent work, amidst COVID-19, Chung et al. (2020) investigated online learning readiness among university students. The study examined whether demographic factors caused differences in the participants’ willingness for online learning experiences. It also explored their preferred online learning methods and the challenges they faced. The data collected from 399 students at the University of Malaysia revealed that most participants were ready for virtual learning. Nevertheless, degree students were more prepared than those who held a diploma; females were more inclined than males. Female and degree students were more satisfied with online learning and had superior learning experiences than male and diploma students. More than half of the students did not want to keep on with online education in the future. Most students preferred online learning through pre-recorded lectures uploaded to Google Classroom and YouTube. The most considerable challenge for degree students was the internet connectivity; however, the main problem was understanding the content for diploma students.

However, a literature review indicates that no previous studies have investigated learners’ affection, perceptions, and problems during the COVID-19 pandemic and have not considered participants’ resiliency and self-image as factors that may affect their views on such courses. Thus, the researchers of the present research designed a sequential exploratory mixed methods study. According to Creswell (2015), the design aims to “explore a problem through qualitative data collection and analysis, develop an instrument or intervention, and follow with a third quantitative phase” (p. 39). The researchers followed three phases in the current study. The first phase was devoted to gathering qualitative data to develop a questionnaire to uncover respondents’ resiliency and self-image and address their affection, perceptions, and problems in online classes. In the second phase, the newly-developed questionnaire was validated via factorial analysis. In the third phase, it was administered, and the results were analyzed. The findings were triangulated by obtaining data from the students who had volunteered to participate in semi-structured interviews, which could help researchers expand their understanding of the variables under scrutiny. The following research questions addressed the issues:

1. To what extent are Iranian university students resilient and have a positive self-image?
2. What are Iranian university students’ responses to the newly-developed questionnaire regarding affection, perceptions, and problems toward virtual classes?
3. How do the interviewees view online courses?

## Method

### Participants

In the first phase, three university instructors with more than ten years of teaching experience helped the researchers to find and evaluate items for a questionnaire. They reviewed the entries for clarity and appropriateness. They also reviewed the interview questions. Besides, 26 university students with similar characteristics to the target sample population verified the clarity and suitability of the extracted items for the questionnaire. Five of them stated their opinions about the interview questions.

In the second phase, 280 Iranian students selected from various academic settings in Iran participated. They were 182 females and 98 males majoring in Translation Studies and TEFL at different levels of language proficiency, with their ages ranging from 20 to 47 years old. The participant pool consisted of 100 BA, 136 MA, and 44 Ph.D. Iranian university students. They were selected non-randomly based on convenience sampling. Their answers to a newly-developed questionnaire helped the researchers to validate the instrument.

In the third phase, 252 Iranian students majoring in Translation Studies and TEFL answered the validated virtual class Affection, Perceptions, Problems, Resiliency, and Self-image Questionnaire (APPRSQ). The respondents were 174 females and 78 males majoring in Translation Studies and TEFL, at different levels of language proficiency, within the age range of 19 and 47 years old. The analysis of their demographic information showed that 82 BA, 131 MA, and 39 Ph.D. candidates had responded to the questionnaire. Convenience sampling was the method of sample selection.

Additionally, 20 students who answered (APPRSQ) volunteered to participate in the online interview sessions via WhatsApp to help the researchers triangulate the data. They were females (n = 16) and males (n = 4) studying at BA and MA levels and were majoring in TEFL and Translation Studies.

### Instruments

The first data gathering tool was the validated virtual class APPRSQ used to collect the required data. The newly-developed questionnaire consisted of two sections: the first section asked for the participants’ demographic information. The second section consisted of 37 items: items 1–16 targeted the respondents’ resiliency and self-image, and items 17–37 explored affection, perceptions, and problems in virtual learning during the pandemic on a five-point Likert scale from “Strongly Disagree to “Strongly Agree” (Appendix 1). Understanding the participants’ resiliency and self-image could help the researchers have a clear picture of the respondents’ characteristics and interpret the data obtained from the items on affection, perceptions, and problems of virtual classes.

Another tool for data collection was a semi-structured interview conducted after the administration of APPRSQ to triangulate the data and attain an in-depth understanding “of the phenomenon under investigation” (Rashtchi & Birjandi, 2018, p.143). After consulting with three experts and five students whose characteristics were close to the study participants, the researchers formulated the interview questions. The interviews were recorded and analyzed via a deductive approach to enable the researchers to explore the students’ viewpoints in virtual classes (Appendix 2). The researchers employed the deductive approach to align with the research questions. As Bingham and Witkowsky (2022) state, the deductive approach uses predetermined codes for classifying the data. Therefore, the researchers first focused on the five factors of APPRSQ. Then they reviewed the data and categorized them under the broad themes related to the study’s primary concern.

### Procedure

#### The first phase

##### Developing the questionnaire (APPRSQ)

Firstly, the researchers reviewed the related literature to follow the standard criteria for developing a reliable and valid questionnaire (Dörnyei, 2003). They found 97 concepts related to the issues under examination. Then three experts in the field were asked to review the items. The researchers analyzed the experts’ opinions and extracted the themes that overlapped with the 97 initial items. After revising, 80 items remained. The three experts reviewed the entries for redundancy, clarity, and content—the purpose was to remove the vague and irrelevant items, which led to decreasing the questionnaire to 57 items.

The revised 57-item questionnaire was content validated with 26 students (similar to the target sample) for additional feedback on the clarity and appropriateness. Then the researchers gathered more information concerning the items by asking the respondents to explain their answers to ensure no discrepancy between the questions’ intended meaning and the respondents’ understanding. The researchers examined the items for clarity, readability, and redundancy which resulted in the removal of 20 items.

#### The second phase

##### Validating the questionnaire (APPRSQ)

The 37-item questionnaire was administered online to 280 Iranian university students. The responses were entered into SPSS for examining the questionnaire’s construct validity and reliability. The reliability of the newly-developed questionnaire was calculated using Cronbach’s alpha coefficient. Exploratory Factor Analysis was run to investigate the construct validity of the instrument. The results of the factorial analysis led to retaining all of the items. The steps followed to validate the questionnaire are presented in the Results section.

#### The third phase

##### Administering the questionnaire (APPRSQ)

After the validation process, APPRSQ was administered online, and of the 278 questionnaires, 252 were complete and thus could be considered for further analysis. The procedure of data collection took six months and a half.

At the final stage, the researchers explored to what extent the findings obtained from APPRSQ were compatible with participants’ answers to the interview questions. Due to the pandemic, the interviews were performed via WhatsApp. Each recording interview took about ten minutes, during which the respondents stated their opinions about virtual classes.

## Results

### Validating APPRSQ

In the first phase, the researchers had to look into the underlying structural dimensions of the questionnaire. Therefore, Exploratory Factor Analysis (EFA) was performed. The Kaiser–Meyer–Olkin measure of sampling adequacy (KMO) value and Bartlett’s test were examined to verify that the data were suitable for factor analysis (Table 1). KMO value turned out to be 0.93, which is beyond 0.60, and Bartlett’s test was significant (*p* < 0.001). Therefore, the results of factor analysis were found to be appropriate for the existing data set.

**Table 1:** KMO & Bartlett’s test

Kaiser–Meyer–Olkin measure of sampling adequacy	.931
Bartlett’s Test of Sphericity	
9019.461	1158.584
666	300
.000	.000

A factor analysis through Varimax rotation was conducted on the underlying construct of the questionnaire. SPSS extracted five factors, with eigenvalues of more than 1, explaining 72.04% of the variance. Five-point Likert scales were used for each item, ranging from 1 (Strongly Disagree) to 5 (Strongly Agree) (Table 2).

**Table 2:** Principal component analysis on APPRSQ

Component	Initial eigenvalues			Extraction sums of squared loadings			Rotation sums of squared loadings^a^
	Total	% of variance	Cumulative %	Total	% of variance	Cumulative %	Total
1	14.523	39.251	39.251	14.523	39.251	39.251	11.598
2	6.904	18.659	57.910	6.904	18.659	57.910	7.664
3	2.545	6.877	64.787	2.545	6.877	64.787	4.364
4	1.535	4.149	68.936	1.535	4.149	68.936	9.644
5	1.148	3.102	72.038	1.148	3.102	72.038	8.433
6	.846	2.285	74.323				
7	.746	2.016	76.339				
8	.717	1.937	78.277				
9	.621	1.680	79.956				
10	.601	1.624	81.580				
11	.563	1.521	83.101				
12	.525	1.418	84.519				
13	.492	1.329	85.848				
14	.459	1.242	87.090				
15	.421	1.139	88.229				
16	.356	.961	89.190				
17	.344	.929	90.118				
18	.331	.894	91.012				
19	.303	.819	91.831				
20	.293	.793	92.623				
21	.281	.758	93.382				
22	.273	.737	94.119				
23	.265	.717	94.836				
24	.227	.613	95.449				
25	.209	.564	96.013				
26	.200	.540	96.553				
27	.184	.498	97.051				
28	.168	.455	97.506				
29	.148	.401	97.906				
30	.135	.366	98.272				
31	.124	.335	98.607				
32	.121	.326	98.933				
33	.105	.285	99.218				
34	.095	.257	99.475				
35	.079	.213	99.688				
36	.072	.194	99.882				
37	.044	.118	100.000				

Extraction method: principal component analysis

^a^When components are correlated, sums of squared loadings cannot be added to obtain a total variance

Table 3 contains the initial commonalities before rotation. As observable from Table 3, all commonalities are high (> 0.30) and acceptable. The communality values for the developed questionnaire ranged from 0.33 to 0.87.

**Table 3:** Initial communality values in PCA

Item	Initial	Extraction
Item1	1.000	0.755
Item2	1.000	0.725
Item3	1.000	0.779
Item4	1.000	0.736
Item5	1.000	0.714
Item6	1.000	0.617
Item7	1.000	0.719
Item8	1.000	0.757
Item9	1.000	0.702
Item10	1.000	0.648
Item11	1.000	0.583
Item12	1.000	0.763
Item13	1.000	0.719
Item14	1.000	0.610
Item15	1.000	0.758
Item16	1.000	0.751
Item17	1.000	0.813
Item18	1.000	0.661
Item19	1.000	0.644
Item20	1.000	0.578
Item21	1.000	0.765
Item22	1.000	0.673
Item23	1.000	0.598
Item24	1.000	0.834
Item25	1.000	0.802
Item26	1.000	0.839
Item27	1.000	0.820
Item28	1.000	0.845
Item29	1.000	0.742
Item30	1.000	0.795
Item31	1.000	0.330
Item32	1.000	0.581
Item33	1.000	0.700
Item34	1.000	0.816
Item35	1.000	0.781
Item36	1.000	0.872
Item37	1.000	0.829

Extraction method: principal component analysis

As Table 4 shows, five factors loaded after rotating the factors in Principal Component Analysis (PCA). Items loading above 0.40 were considered acceptable. In this phase, all items were acceptably loaded on five factors. As Table 4 shows, ten items that have their highest loading from factor 1 are listed from the highest (item 35) to the lowest (item 27); in factor 2, nine items (highest: item 12, lowest: item 8), in factor 3 five items (highest: item 30, lowest: item 31), in factor 4 seven items (highest: item 4, lowest: item 14), and finally in factor 5 six items (highest: item 21, lowest: item 19) are listed.

**Table 4:** Rotated factor matrix^a^ in PCA

Items	Component				
	1	2	3	4	5
Item35	− .915				
Item34	− .885				
Item28	− .885				
Item37	− .880				
Item36	− .875				
Item26	.871				
Item24	.852				
Item25	.846				
Item17	.812				
Item27	.771				
Item12		.857			
Item10		.823			
Item13		.819			
Item7		.673			
Item5		.669			
Item9		.491		.423	
Item11		.489		.397	
Item6		.487		.346	
Item8		.448		.443	
Item30			.891		
Item33			.839		
Item29			.819		
Item33			.593		
Item31			.523		
Item4				.855	
Item16				.835	
Item3				.832	
Item1				.782	
Item15				.772	
Item2				.733	
Item14				.520	
Item21					.873
Item22					.729
Item23					.670
Item18					.599
Item20					.544
Item19					.487

Extraction method: principal component analysis

Rotation Method: Oblimin with Kaiser Normalization^a^

^a^Rotation converged in 17 iterations

**Fig. 1:**
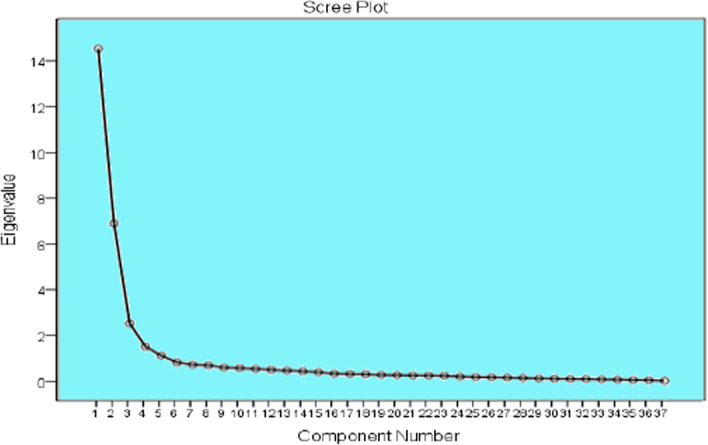
Scree plot for APPRSQ in PCA

As given in the scree plot in Fig. 1, there is a clear break between the first and second and the second and third components. Furthermore, the scree plot indicates another little break after the fifth component. Therefore, extracting five components was legitimate.

Following the rotation of each of the items on the five components, the items loading on the five factors with ten items loading above 0.3 on component one, nine items loading on component two, five items on component three, seven items on component four, and six items on component five were found (Table 5). The Exploratory Factor Analysis (EFA) results and their pertinent reliability indices are also shown in Table 5. As the results indicate, those factors and their developed items were confirmed, suggesting that APPRSQ is a valid and reliable tool for data gathering purposes.

**Table 5:** Five factors of APPRSQ with related reliability indices

Factor	No. of items	Cronbach’s alpha
1. Resiliency (items 1, 2, 3, 4, 14, 15, 16)	10	0.933
2. Self-image (items 5, 6, 7, 8, 9, 10, 11, 12, 13)	9	0.905
3. Affection (items 18, 19, 20, 21, 22, 23)	6	0.802
4. Perceptions (items 17, 24, 25, 26, 27, 28, 34, 35, 36, 37)	10	0.948
5. Virtual class problems (items 29, 30, 31, 32, 33)	5	0.852
Total questionnaire	37	0.894

As the final step, parallel analysis was performed to ensure that the five components extracted from EFA could be retained. Watkins’ (2006) method, which compares the initial eigenvalues computed with the SPSS software against the ones simulated one hundred times, was used to determine how many factors to extract. The factors whose observed eigenvalues were higher than the simulated ones were kept. As displayed in Table 6, the first five observed eigenvalues were larger than the simulated ones. Thus, the parallel analysis suggested five factors to be extracted as underlying constructs of the 37 items of the questionnaire.

**Table 6:** Watkins’ parallel analysis

	Observed	Simulated	Decision
1	14.523	1.806	KEEP
2	6.904	1.709	KEEP
3	2.545	1.628	KEEP
4	1.535	1.514	KEEP
5	1.148	1.109	KEEP
6	0.846	1.055	DROP
7	0.746	1.003	DROP
8	0.717	0.956	DROP
9	0.621	0.914	DROP
10	0.601	0.871	DROP
11	0.563	0.834	DROP
12	0.525	0.796	DROP
13	0.492	0.760	DROP
14	0.459	0.724	DROP
15	0.421	0.688	DROP
16	0.356	0.656	DROP
17	0.344	0.620	DROP
18	0.331	0.586	DROP
19	0.303	0.557	DROP
20	0.293	0.526	DROP
21	0.281	0.495	DROP
22	0.273	0.462	DROP
23	0.265	0.433	DROP
24	0.227	0.402	DROP
25	0.209	0.374	DROP
26	0.2	0.347	DROP
27	0.184	0.319	DROP
28	0.168	0.290	DROP
29	0.148	0.263	DROP
30	0.135	0.235	DROP
31	0.124	0.209	DROP
32	0.121	0.178	DROP
33	0.105	0.151	DROP
34	0.095	0.120	DROP
35	0.079	0.089	DROP
36	0.072	0.055	DROP
37	0.044	0.014	DROP

### Phase two: results of APPRSQ

The researchers answered the research questions by dividing APPRSQ into five components. The participants’ responses to each section were analyzed. All tables present the frequencies (f), percentages, and mean scores (M) obtained from the respondents’ answers.

#### First research question

Tables 7 and 8 provide answers to the first research question. As Table 7 shows, items 1, 2, 3, 4, 14, 15, and 16 address the respondents’ resiliency. One hundred and twenty-five (out of 252 participants) selected “Strongly Agree” and “Agree” (SA & A) to item 14: “Difficult experiences in life make me stronger,” which comprise 59.9 percent of the answers (M = 3.57). The next most agreed item is 2: “I adapt quickly to new developments” (49.6%, M = 3.23). Item 16: “I can tolerate high levels of ambiguity and uncertainty about situations,” which is the lowest agreed-upon item (41.7%, M = 3.05), followed by item 4: “Feelings of anger, loss, and discouragement do not last long” (41.3%, M = 3.10) also seem to receive relatively high degrees of agreement. The researchers believe the respondents’ answers to resiliency indicate that, in general, Iranian university students (majoring in Translation Studies and TEFL) are almost resilient and can cope with changes they encounter in their lives.

**Table 7:** Frequency, percentage, & mean score of responses to APPRSQ (resiliency)

Item	SD & D		N		SA & A		M
	f	%	f	%	f	%	
*Resiliency*							
1. In a crisis or chaotic situation, I calm myself and focus on taking useful actions	91	36.1	42	17	119	47.2	3.1
2. I adapt quickly to new developments	71	28.2	56	22	125	49.6	3.23
3. I can recover emotionally from losses and setbacks	76	30.2	54	21	122	48.4	3.20
4. Feelings of anger, loss, and discouragement do not last long	86	34.1	62	25	104	41.3	3.10
14. Difficult experiences in life make me stronger	51	20.2	35	14	166	59.9	3.57
15. I can convert misfortune into good luck and find benefits in bad experiences	81	32.1	53	21	118	46.8	3.15
16. I can tolerate high levels of ambiguity and uncertainty about situations	88	34.9	59	23	105	41.7	3.05

**Table 8:** Frequency, percentage, & mean scores rf responses to APPRSQ (self-image)

Item	SD & D		N		SA & A		M
	f	%	f	%	f	%	
*Self-image*							
5. I consider myself self-confident and mostly appreciate what I have	33	13.1	18	7	201	79.8	3.92
6. I learn valuable lessons from my and others’ experiences	47	18.7	70	28	135	53.6	3.41
7. I am good at solving problems	41	16.3	27	11	184	73.0	3.71
8. I can manage life complexities well	39	15.5	56	22	157	62.3	3.60
9. I can act more effectively when I am free to do what I think is best in every situation	43	17.1	60	24	149	59.1	3.48
10. I am a good listener and have good empathy skills	27	10.7	33	13	192	76.2	3.88
11. Generally speaking, I consider myself a non-judgmental person in life	44	17.5	70	28	138	54.8	3.46
12. I am good at cooperating and working with others	37	14.7	19	8	196	77.8	3.85
13. I have an independent outlook on life	29	11.5	21	8	202	80.2	4.01

As indicated in Table 8, items 5, 6, 7, 8, 9, 10, 11, 12, and 13 explored how the respondents viewed themselves. For instance, item 13: “I have an independent outlook on life,” is the highest agreed-upon item (80.2%, M = 4.01), followed by number 5: “I consider myself self-confident and mostly appreciate what I have” (79.8%, M = 3.92). However, item 6: “I learn valuable lessons from my and others’ experiences.” is the lowest agreed-upon (53.6%, M = 3.41), followed by 11: “Generally speaking, I consider myself a non-judgmental person in life” (54.8%, M = 3.46). The researchers concluded that the participants had a positive image of themselves. They viewed themselves as independent, self-confident, and satisfied with their lives.

#### Second research question

Tables 9, 10, and 11 answer the second research question. As Table 9 shows, items 18, 19, 20, 21, 22, and 23 addressed the respondents’ affection for virtual classes. Item 23: “Student-based strategies are highlighted in virtual classes” (65.1%, M = 3.59) is the highest agreed upon, followed by number 22: “I believe virtual classes are the best substitute during the pandemic outbreak” (61.5%, M = 3.63). Nevertheless, item 16: “I like virtual classes because I do not need to leave my house (53.2%, M = 3.24), followed by 19: “Virtual learning helps me develop my knowledge of computers and the internet” (50.8%, M = 3.10) are the least agreed upon items. As the results reveal, the students who participated in the present study believed that such classes were a good option during the COVID-19 outbreak. Their responses to items 19, 21, and 23 show that they have to rely more on themselves than be dependent on their teachers in virtual classes. About half of the respondents thought such classes could boost their digital knowledge (item 19) and be time-saving (items 18 and 20).

**Table 9:** Frequency, percentage, & mean score for responses to APPRSQ (affection)

Item	SD & D		N		SA & A		M
	f	%	f	%	f	%	
*Affection*							
18. I like virtual classes because I do not need to leave my house	90	35.7	28	11	134	53.2	3.24
19. Virtual learning helps me develop my knowledge of computers and the internet	54	21.4	70	28	128	50.8	3.37
20. I can manage my time in virtual classes better than in face-to-face ones	68	27.0	41	16	143	56.7	3.41
21. Virtual learning helps me do more research on the net regarding my class syllabus	33	13.1	67	27	152	60.3	3.56
22. I believe virtual classes are the best substitute during the pandemic outbreak	33	13.1	64	25	155	61.5	3.63
23. Student-based strategies are highlighted in virtual classes	48	19.0	40	16	164	65.1	3.59

**Table 10:** Frequency, percentage, & mean scores of responses to APPRSQ (perceptions)

Item	SD & D		N		SA & A		M
	f	%	f	%	f	%	
*Perceptions*							
17. I find virtual classes interesting and useful	141	56.0	34	13	77	30.6	2.68
24. The virtual system is user-friendly	147	58.3	36	14	69	27.4	2.51
25. I am satisfied with the online interactions with my instructor	145	57.5	38	15	69	27.4	2.50
26. I am satisfied with the online interactions with my classmates	149	59.1	39	15	64	25.4	2.40
27. I believe students learn better through virtual classes	153	60.7	38	15	61	24.2	2.42
28. Virtual classes are difficult to handle and, therefore, frustrating to use	90	35.7	23	9	139	55.2	3.34
34. I believe virtual classes might be more of a distraction than an aid to my learning process	86	34.1	42	17	124	49.2	3.29
35. I believe attending virtual classes is energy-and-time consuming	87	34.5	38	15	127	50.4	3.29
36. I feel confused or disoriented in virtual classes	80	31.7	32	13	140	55.6	3.44
37. I find the virtual system difficult to use	91	36.1	35	14	126	50.0	3.21

**Table 11:** Frequency, percentage, & mean score for students’ responses to APPRSQ (virtual class problems)

Item	SD & D		N		SA & A		M
	f	%	f	%	f	%	
*Virtual class problems*							
29. Slow internet connection is a major problem in virtual classes	14	5.6%	9	4%	229	90.9%	4.50
30. Attending virtual classes requires having an appropriate device like laptops, personal computers, or smartphones	8	3.2%	5	2%	239	94.8%	4.66
31. Having technical skills in a computer can facilitate the quality of my learning in virtual classes	9	3.6%	23	9%	220	87.3%	4.15
32. Downloading errors, installation problems, and audio and video problems impede learning	16	6.3%	20	8%	216	85.7%	4.37
33. If I do not have laptops, personal computers, or smartphones, I am deprived of learning opportunities	14	5.6%	9	4%	229	90.9%	4.56

Items 17, 24, 25, 26, 27, 28, 34, 35, 36, and 37, as indicated in Table 10, addressed the respondents’ perceptions regarding virtual classes. Item 36: “I feel confused or disoriented in virtual classes” is the highest agreed-upon (55.6%, M = 3.44), followed by 28, “Virtual classes are difficult to handle and, therefore, frustrating to use” (55.2%, M = 3.34). However, Item 27: “I believe students learn better through virtual classes,” is the lowest agreed-upon item (24%, M = 2.42), followed by item 26: “I am satisfied with the online interactions with my classmates.” (25%, M = 2.40).

As Table 11 shows, items 29, 30, 31, 32, and 33 addressed the problems of virtual classes. Item 30: “Attending virtual classes requires having an appropriate device like laptops, personal computers, or smartphones” is the highest agreed upon (94.8%, M = 4.66), followed by 33: “If I do not have laptops, personal computers, or smartphones, I have to borrow to attend the class” (90.9%, M = 4.56). While item 31: “Having technical skills in a computer can facilitate the quality of my learning in virtual classes.” is the lowest agreed-upon (87.3%, M = 4.15), followed by item 32: “Virtual classes cannot be beneficial for all courses” (85.7%, M = 4.37). Overall, the results indicated that the Iranian Translation and TEFL-major university students confront problems with virtual classes. Slow internet connection, lack of electronic devices, and lack of adequate technical skills are barriers to their satisfaction with online courses. The researchers of the present study believe that such problems might decrease the practicality of virtual classes.

**Fig. 2:**
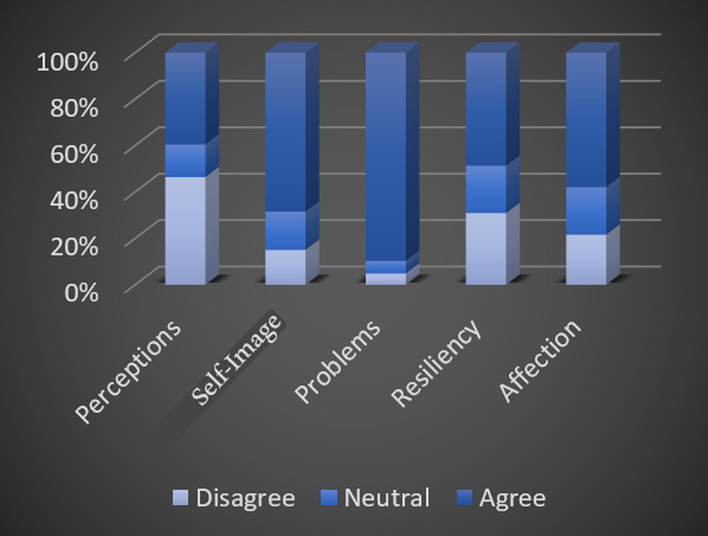
Percentage of responses to APPRSQ

Figure 2 shows the percentages of the participants’ responses (Strongly Agree and Agree; neutral; Disagree and Strongly Disagree) to the different components of APPRSQ.

#### Third research question

The third research question dealt with the interviews through which the researchers attempted to provide a deeper understanding of online classes. The researchers focused on the themes and organized data following the research questions (deductive approach).

*1. Resilience* The first theme the researchers extracted could be classified under resilience. The interviewees believed they could adapt to the new situation and rely on themselves in online courses. They thought that virtual classes were a unique experience during their education. Practicing in online courses could help them cope with the challenges they might face in their lives.

“I learned to be more resilient through difficulties during these classes…we have to help each other overcome the imposing situation.”

“I am experiencing a new type of learning. I have to adapt to it and the modern world. The classes push me to rely on myself.”

“I think the classes push me to be like an autonomous learner.”

“I think online classes help me use time effectively. I can study at my own pace. I feel more confident and perform better in virtual classes than in regular classes.”

*2. Self-image* The researchers could detect respondents’ views about themselves while expressing their ideas about online classes. These assertions could be attributed to self-image, another factor in the present study. The interviewees believed that the classes could enhance their self-confidence and help them listen more carefully to people. They stated that they learned to evaluate people’s views rather than judge them. Not having face-to-face contact with the teacher and classmates enhanced their attention. The challenges in online courses helped them view learning as a problem-solving activity.

“Online classes helped me feel stronger and face my learning problems with more confidence.”

“Although I don’t like online classes very much, I think I am more attentive in class because I don’t want to miss any point.”

“I feel stronger in online classes, listen to people, express my ideas, and be fair and not judge classmates.”

*3. Affection* The third theme the researchers could extract was students’ affection toward virtual classes. In general, the interviewees were positive toward adopting virtual classes in the sense that such classes were the only best substitute for regular classes. They believed such a shift was inevitable. Thus, they tried to benefit from the classes and look at them from an optimistic viewpoint. However, some interviewees stated their preference for face-to-face classes and maintained that the best way for learning is by interacting and socializing with teachers and peers.

“Online classes are time-saving.”

“It’s time to improve my technological knowledge.”

“At the moment, there is no better option, or education will collapse.”

“Although [the classes] will not replace regular classes, they help improve on my own, learn internet search, practice extra reading to catch up with the class content.”

*4 Perception* The interviewees asserted that although they tried to cope with virtual classes, they generally preferred face-to-face courses. The interviewees stated that the classes seemed interesting to them; however, after some time, they felt tired and liked to get back to regular classes, socialize with classmates, and interact with instructors. They felt that being in contact with instructors and classmates could foster learning, increase motivation to learn, and decrease anxiety. The interviewees’ answers verified the participants’ responses to APPRSQ, indicative of preferring regular classes to virtual ones.

“I think the most important factor is interaction, the lack of which is bolded in these classes…so it might decrease my motivation.”

“I miss my teachers… virtual classes can never replace face-to-face classes…may be an acceptable substitute during the pandemic.”

“Isolation bothers me a lot. The lack of face-to-face interaction affects my learning.”

“Virtual class is somehow not fair…because there is not any distinct line to show your real performance.”

*5-Problems* The interviewees believed technological illiteracy was a significant deficiency in online classes. The sudden transference to such classes showed that neither students nor teachers had adequate information on running and handling online courses. However, the interviewees stated that the pandemic provided the opportunity for learning how to act in online classes:

“I think students need some familiarity with the virtual environment…because the sudden change due to the lack of online education before the pandemic is more than expected. Students’ illiteracy in technology is a significant problem.”

“Using the social media does not give literacy, and students’ knowledge in this regard is very basic.”

“Online classes could boost students’ and teachers’ knowledge of computers technology.”

The respondents’ main complaint was the slow internet connection. They thought internet disconnection and lack of video connection increased their anxiety. Besides, the problems they encountered in using the software in the classes frustrated them. They emphasized “interactive option” as the most vital characteristic of any software used in virtual learning.

“Slow internet connection is the major problem with online classes. Without a consistent connection. There can be a lack of continuity in the learning and teaching process.”

“I think the online domain should allow interaction between the teacher and students. Connecting with the teacher and peers is very important.”

Another problem was the teachers’ teaching style. The respondents believed that a change in teachers’ professional behaviors in online classes could contribute to the fruitfulness of such courses. Being more energetic, interactive, avoiding monotonous speeches, and having a positive attitude can reduce learners’ anxiety, motivate them, and foster learning.

“Face-to-face classes gave me a reason to talk, but virtual classes with a monotonous voice of our teacher make me tired and reduce my motivation.”

“Teachers should consider revising their teaching styles. Reading out from the book and not encouraging learners’ participation make the classes dull.”

Table 12 shows the themes with the highest frequency of occurrence in the interviewees’ responses.

**Table 12:** Themes with highest frequency as stated by interviewees

Categories	No. of students (n = 20)	Percentage
1. Time-saving	19	95
2. Flexibility in place	19	95
3. Self-regulation	13	65
4. Variety in teaching tools	14	70
5. Instill self-confidence	13	65
6. Slow internet connection	18	90
7. Technological illiteracy	15	75
8. Knowledge illiteracy	12	60
9. Cheating	14	70
10. Lack of teachers’ monitoring	15	75
11. Lack of interaction	16	80
12. Sense of isolation	15	75

## Discussion

Virtual classes forced students to welcome and look at online learning as a solution to deal with the regulations of the lockdown during the pandemic. However, what learners think and feel about the type of teaching–learning such courses provide is still unclear. Studying such classes from students’ perspectives seems vital in realizing the extent to which the education system can benefit or suffer from online learning. The current study attempted to develop and validate a self-report questionnaire (APPRSQ) and examine how Iranian university students viewed virtual classes. Their answers were more reliable when researchers could have information about the participants’ resiliency and how they perceived themselves (self-image). The results of the factorial analysis indicated that APPRSQ was a valid and reliable tool for data gathering and valuable for future studies.

Regarding the first research question addressing students’ resiliency and self-image, the researchers infer that the participants could stand against the changes they faced in the education system from face-to-face to online learning after the pandemic. The researchers assume that students’ resiliency can be due to the history of the Iranian nation and how they have survived different events like wars and occupations during their country’s long history, a discussion of which is beyond the scope of the present study. Therefore, it could be concluded that the participants had an acceptable resiliency level to tolerate virtual classes. Accordingly, the students could adapt to the new situation despite the challenges it had for them (Rodríguez-Fernández et al., 2018).

As Waxman et al. (2002) argue, resilience is the reason for success in education and helps students to be successful in academic settings. Resilient students can tolerate risks and adjust to the shift toward online teaching and learning caused by the COVID-19 pandemic. The abrupt change to virtual learning did not endanger their well-being (Al-Rabiaan et al., 2020) and thus did not affect the authenticity of their answers to APPRSQ.

Besides, the Translation Studies and TEFL students who took part in the current study had a constructive image of themselves. They were independent, self-confident, and believed in themselves. Then again, the present study’s researchers infer that although their views toward virtual classes were not entirely positive, this view was not due to their perceived image of themselves. They were not pessimistic toward themselves or life. This aspect of the APPRSQ was necessary because how individuals view themselves can affect their perceptions and opinions (Waxman et al., 2002).

Learners’ affection was one of the variables considered in evaluating virtual courses in the present study. According to Iozzi (1989), focusing on the affective domain is helpful because it deals with positive environmental attitudes and values. Positive affective factors accelerate cognition (Brophy, 2008) and thus boost learning. The participants’ answers to the items which addressed affection indicated that they had positive feelings toward online learning and thought it was the best substitute for face-to-face classes during the COVID-19 lockdown. Their answers revealed that they had realized the necessity to rely on themselves and their cognitive capabilities to become autonomous learners (Hall, 2011). Online learning encouraged the participants to get involved in learning and think about themselves and their learning process (Aryanjam et al., 2021). The participants believed that online courses facilitated learning about technological issues, a finding also emphasized by the interviewees’ responses. The answers to APPRSQ and interview questions also showed that the participants were emotionally satisfied with online learning; however, they preferred regular classes due to the lack of interaction and social contact with peers and teachers.

Furthermore, regarding the participants’ perceptions, the researchers’ analysis of the participants’ responses showed that they had different views and thoughts about online learning, probably due to differences in learning styles and personal preferences arising from how they cognitively perceived online education (Eforn, 1969). The surveys on learners’ attitudes and perceptions regarding online learning before and after the COVID-19 breakdown portray this conclusion. There seems to be no unanimous agreement among learners regarding the advantages of such classes. In other words, in many studies similar to the current one, many students preferred online courses, although many others disapproved of them (e.g., Bączek et al., 2021; Ituma, 2011; Thomas et al., 2020). However, in most studies, slow internet connection and technical issues formed the prime complaint of the respondents that affected their perceptions and led them not to approve of the practicality of online learning (e.g., Agung et al., 2020; Bączek et al., 2021; Bao, 2020; Keller & Cernerud, 2002).

The problems of virtual classes were the next issue addressed in the study. The interviews showed that engagement and participation in teaching–learning activities were significant challenges for the learners. As Singh and Thurman (2019) argue, the move from offline to online mode causes a considerable challenge since direct communication is lost in virtual settings, and communication between students and the teacher is blurred. Such factors could be the underlying source of many complaints. However, the participants’ primary problems were the slow internet connection and lack of equipment needed. Besides, inadequate knowledge of working in digital environments prevented the participants from benefitting from online courses. The interview results revealed that teachers should change their teaching behaviors and adopt techniques to lead vigorous classes.

The findings of the study align with some previous studies. For example, Abbasi et al. (2020) found that the majority of the participants had a negative perception of virtual classes and did not believe in the usefulness of such courses. Their study indicated the superiority of face-to-face learning over e-learning. Agung et al. (2020) also found that Indonesian students were not ready for online classes due to technical problems (e.g., internet connection), inaccessibility of teaching platforms, and inability to connect to media. Similarly, Bączek et al. (2021) found that medical students believed face-to-face classes were more interactive than virtual courses. However, Ituma (2011) found that most students had positive perceptions of e-learning, contrasting with the current survey.

All in all, researchers of the current study infer that the participants could adapt to the new situation (online teaching) imposed on them by the pandemic and could use online courses as an opportunity (Rodríguez-Fernández et al., 2018). In other words, despite the adversity due to the challenges in online classes, the students could find positive points in such courses and somehow like them. This finding partially contrasts with Valizadeh (2021), who found that Turkish students experienced anxiety during online classes due to situation-specific measures (Heydarpour Meymeh et al., 2019).

## Conclusion

This study contributed to the appraisal of virtual learning and tried to look into issues that might concern educational institutions, authorities, and society about the strengths and weaknesses of virtual classes. The researchers hope the study will help the authorities to view virtual courses from students’ perspectives and promote their quality. Accordingly, this study gives practitioners information about how university students view virtual classes. Education authorities should allocate sufficient support concerning the problems clarified by the respondents. Educational institutions should maintain a direct and constant link to a technical support team to save learners from minimizing breakdowns and inaccessibility to upgrade efficiency in virtual classes. Educational institutions should give intensive attention to their infrastructure by upgrading and modernizing the virtual system, particularly the hardware and servers, to deal with students’ confusion in virtual classes.

Moreover, students need training and preparation before participating in online classes. They also need training in computer skills. Therefore, mandatory and intensive training courses need to be offered to students. This study can bridge the gap in the literature by examining university students’ perspectives on virtual learning and might be used as a foundation to open the door for similar studies in the future. Therefore, this study contributes to further research regarding virtual learning.

## Data Availability

The data are available and can be accessed by other researchers upon request.

## Appendix 1

### Virtual Class Affection, Perceptions, Problems, Resiliency, and Self-image Questionnaire (APPRSQ)

**Table:**

**1. Gender**	1. Male	2. Female	
**2. Major**	**1. TEFL**	**2. Translation Studies**	
3. Level of Education	1. BA	2. MA	3. Ph.D
4. Age			

**Guideline:** Please read the following statements and tick the number that shows how you think about the new system caused by Covid-19-Virtual classes.

SD = Strongly Disagree

D = Disagree

N = Neutral

A = Agree

SA = Strongly Agree

**Table:**

Item	SD	D	N	A	SA
	1	2	3	4	5
1- In a crisis or chaotic situation, I calm myself and focus on taking useful actions	1	2	3	4	5
2- I adapt quickly to new developments	1	2	3	4	5
3- I can recover emotionally from losses and setbacks	1	2	3	4	5
4- Feelings of anger, loss, and discouragement do not last long	1	2	3	4	5
5- I consider myself self-confident and mostly appreciate what I have	1	2	3	4	5
6- I learn valuable lessons from my and others’ experiences	1	2	3	4	5
7- I am good at solving problems	1	2	3	4	5
8- I can manage life complexities well	1	2	3	4	5
9- I can act more effectively when I am free to do what I think is best in every situation	1	2	3	4	5
10- I am a good listener and have good empathy skills	1	2	3	4	5
11- Generally speaking, I consider myself a non-judgmental person in life	1	2	3	4	5
12- I am good at cooperating and working with others	1	2	3	4	5
13- I have an independent outlook on life	1	2	3	4	5
14- Difficult experiences in life make me stronger	1	2	3	4	5
15- I can convert misfortune into good luck and find benefits in bad experiences	1	2	3	4	5
16- I can tolerate high levels of ambiguity and uncertainty about situations	1	2	3	4	5
17- I find virtual classes interesting and useful	1	2	3	4	5
18- I like virtual classes because I do not need to leave my house	1	2	3	4	5
19-Virtual learning helps me develop my knowledge of computers and the internet	1	2	3	4	5
20- I can manage my time in virtual classes better than in face-to-face ones	1	2	3	4	5
21- Virtual learning helps me do more research on the net regarding my class syllabus	1	2	3	4	5
22- I believe virtual classes are the best substitute during the pandemic outbreak	1	2	3	4	5
23- Student-based strategies are highlighted in virtual classes	1	2	3	4	5
24- The virtual system is user-friendly	1	2	3	4	5
25- I am satisfied with the online interactions with my instructor	1	2	3	4	5
26- I am satisfied with the online interactions with my classmates	1	2	3	4	5
27- I believe the students learn better through virtual classes	1	2	3	4	5
28- Virtual classes are difficult to handle and, therefore, frustrating to use	1	2	3	4	5
29- Slow internet connection is a major problem in virtual classes	1	2	3	4	5
30- Attending virtual classes requires having an appropriate device like laptops, personal computers, or smartphones	1	2	3	4	5
31- Having technical skills in a computer can facilitate the quality of my learning in virtual classes	1	2	3	4	5
32- Downloading errors, installation problems, and audio and video problems impede learning	1	2	3	4	5
33- If I do not have laptops, personal computers, or smartphones, I am deprived of learning opportunities	1	2	3	4	5
34- I believe virtual classes might be more of a distraction than an aid to my learning process	1	2	3	4	5
35- I believe attending virtual classes is energy-and-time consuming	1	2	3	4	5
36- I feel confused or disoriented in virtual classes	1	2	3	4	5
37- I find the virtual system difficult to use	1	2	3	4	5

## Appendix 2

### Interview questions

1. What is your opinion about online classes during the pandemic?
2. What are the merits of online classes?
3. What challenges did you face in online learning?
4. What suggestions do you have for the improvement of online classes?
5. How do you compare virtual and face-to-face classes?
